# Research on Acoustic Environment in the Building of Nursing Homes Based on Sound Preference of the Elderly People: A Case Study in Harbin, China

**DOI:** 10.3389/fpsyg.2021.707457

**Published:** 2021-10-20

**Authors:** Peng Cui, Jun Zhang, Ting Ting Li

**Affiliations:** School of Landscape, Northeast Forestry University, Harbin, China

**Keywords:** acoustic environment, nursing home, elderly's sound perception, elderly's sound preference, acoustic evaluation

## Abstract

Nursing homes are the facilities where the elderly conduct their daily activities. This may lead to a complicated acoustic environment which would potentially affect the ability of the elderly to function. In this study, the main indoor public space of a nursing home in Harbin was taken as the research object, and the methods of field observation, sound measurement, and questionnaire survey were used to explore the sound perception and preference of the elderly. The results revealed that in terms of the temporal and spatial distribution of sound pressure level (SPL), the unit living space had the highest SPL, which was above 60 dB (A). The reverberation time (RT) of the unit living space, medical and health care center corridor, was 2.15 and 2.13 s, respectively, at a frequency of 1,000 Hz, which was within the discomfort range. The results also revealed that an acoustic environment had a strong correlation with humidity and a weak correlation with temperature. However, no significant correlation could be assessed with a luminous environment. The elderly people were generally willing to accept the natural sound sources. The factors of gender and offspring numbers had no significant impact on the evaluation of acoustic environment comfort, whereas marriage and income status affected the comfort. This study may help improve the quality of life of the elderly in the nursing home and provide a reference for the construction and design of pension facilities.

## Introduction

The acoustic environment of care facilities for older adults is garnering widespread scrutiny from both researchers and practitioners due to increasing awareness of geriatric issues and challenges in society. Nursing homes have very peculiar functional patterns, both in terms of space use and daily routines (e.g., recurring activities and sound sources). Each nursing home possesses unique traits and experiences a continuously changing group of users (high turnovers), both in terms of residents and staff members. Assessing the acoustic environments of these facilities might require a multifaceted and more articulate approach than what is commonly deployed for other functional buildings, and tailored solutions will possibly have to be devised for better soundscapes and acoustic environments. The sound environment of nursing facilities affects the physical health of the elderly, such as sleep quality, and also affects their mental health. Studies have shown that different types of elderly people possess different auditory preferences. The elderly with hearing impairment sometimes need greater sound stimulation, and the elderly with a certain amount of cerebellar atrophy is easily averted by noise (Aletta et al., 2017). Therefore, nursing facilities have more stringent acoustic environment requirements than general buildings. It is of great significance to study the acoustic preference characteristics of the elderly in nursing facilities and improve the acoustic environment quality of nursing facilities. Previous studies have focused both on the perception (Kang, 2004; Meng and Kang, 2016) and physical aspects of the acoustic environment of such spaces (Aletta et al., 2017, 2018a; Devos et al., 2018). However, most studies were restrained to the analysis of the optimization of the sound environment in nursing homes or the perception of the sound environment by the elderly. However, the reasons for the difference in acoustic perception among the elderly in the same acoustic environment remain unclear. Little research has been conducted on the influence of individual background differences on the sound perception of the elderly in specific acoustic environments.

Pieter Thomas et al. have made an overview of the literature of sound environment research in nursing homes from 1957 to 2019. The search through the two databases and the additional manual search returned 118 results (Thomas et al., 2020). Among them, there were only four studies that combine the acoustic environment assessment of nursing homes with the difference of the social background of the elderly. Most of the research mainly discussed the optimization of soundscape in nursing homes or the sound perception of the elderly to the existing environment by means of actual measurement. Regarding the sound perception of the elderly, Janus S found that when compared with young people, the elderly are more tolerant and sensitive to sound, and changes in the sound pressure level (SPL) will affect the communication of the elderly and entail serious bodily damage to the elderly when the SPL exceeds 65dB, such as sleep disorder, tinnitus, hypertension, and cardiovascular disease (Janus et al., 2021). Mu et al. (2021) found that the elderly preferred quiet activities and the evaluation of low-decibel (A) and high-decibel (A) activities depended on participation and personal preference. For example, when performing an activity in a public place, participants generally rated sounds more positively than bystanders, and activity sounds associated with music (singing, dancing) were rated being more comfortable than vocal activity sounds (playing chess, playing cards). People have different acoustic perceptions when using different functional spaces (Kawai and Yano, 2002), especially in a socially disadvantaged group. The patients and the elderly have a higher demand for the acoustic environment (Suzuki, 2010; Lin and Lin, 2014; Aletta et al., 2018b).

Acoustic comfort can be defined as the presence of opportunities for acoustic activities that do not annoy others, whereby undesired sound is absent (Thomas et al., 2020). Typical indoor sound sources include fan noise, music, TV, and vocal communication (Torresin et al., 2020). Sound sources are an important factor in sound comfort (Wang et al., 2018; Yi and Kang, 2019). According to the research on indoor acoustics in recent decades, it was found that only Leq A or NC does not suffice to express all the properties present in noise. Therefore, more psycho-acoustic parameters are gradually defined by indoor acoustics researchers. Studies have demonstrated that perception of people of sound in the environment (liking or irritability) depends on the level of Leq A, and on numerous other factors, such as the spectrum characteristics of the sound source, sound volatility, time-varying noise, etc. (Thomas et al., 2020). Thomas et al. (2018) assessed that changing the SPL and controlling the sound source type could effectively improve the psychological pleasure of the elderly. According to Joosse (2011), the sound of the working staff is also an important factor affecting the nursing home acoustic environment, and the noise generated by mechanical equipment can also reduce indoor acoustic environment comfort and increased annoyance (Wu et al., 2012). Another common sound in nursing homes is background music, and studies have shown that both background and foreground music can enhance the appeal of the environment to individuals and boost their levels of happiness (Xie et al., 2020). With an increase in the activities of elderly people and the purchase of new activity equipment in nursing homes sound types have also increased, resulting in more complex acoustic environments. This can cause residents to perceive discomfort in a noisy environment or in an environment devoid of efficient communication. Therefore, as a place with a complex acoustic environment, the sound source and its influence must be systematically studied in the nursing home to improve the acoustic environment of the living indoor space.

Sound preference refers to the preferences of people for sounds. From the perspective of cognitive psychology, people need to comprehend sound and sound events through complete environmental information, not just through mere sounds (Schafer, 1993). Sound has different meanings in different acoustic environments. Tamura found that the majority of people surveyed liked natural sounds such as running water, rain, and birdsong and almost half disliked mechanical sounds (Tamura, 1998). When mechanical sounds are predominant, the degree of relaxation decreases, resulting in reduced acoustic comfort (Wu et al., 2020a). Yang and Kang also stated that in the same environment, acoustic comfort and sound sensitivity levels in women are higher than that of men (Yang and Kang, 2005). Kang further investigated the evaluation of age on acoustic comfort and found that older people prefer the sound of chirping birds (Wang and Kang, 2020). The acoustic environment of nursing facilities possesses the same trend in the sound preferences of the elderly. However, different social backgrounds and experiences lead to disparities in the sound preferences of the elderly (Aletta et al., 2017). The difference of sound source leads to the threshold of ambient sound comfort (Xie et al., 2020).

The objective of this study is to conduct an overall assessment of the acoustic environment in the public space of nursing homes based on the measurement of SPL and reverberation time (RT). Moreover, the study was set to investigate the impact of personal and social factors of elderly people on acoustic environment evaluations and analyze the sound preference of the elderly people in nursing homes. In this study, a typical nursing home including different scales of activity space in Harbin, China, was set as the research site. Objective acoustic parameters, subjective behavioral observations, and questionnaires were used as data-gathering instruments. This study mainly focused on how the personal and social factors of elderly people affect acoustic environment evaluations and the sound preferences of the elderly in nursing homes. The conclusions yielded in this study can provide a reference for the construction and design of facilities for the elderly.

## Methodology

In this present study, relevant data were collected using questionnaires and field measurements, and the credibility of the answers to the questionnaire was enhanced using presurveys and trap questions. The 801 Sound level meter was utilized to record the SPL and calculate the RT. An illuminometer and a microclimate tester were employed to record the indoor luminous and thermal environment, respectively. Furthermore, an HD camera was used to record the activities of the elderly. This research was approved by the ethics review board of the surveyed department, Aixin Nursing Home.

### Survey Site

The definition of nursing homes might vary between countries. For European countries, this should be generally understood as a “public or private facility with a domestic styled environment providing 24 h functional support and care for persons who require assistance and who often have complex health needs and increased vulnerability” (Sanford et al., 2015). For Chinese nursing homes, however, a broader definition of long-term care facility for older adults might apply.

According to a survey, there are 16 nursing homes in Harbin, China. The nursing home selected should meet established standards and be well-equipped, so that the elderly will not be affected by their own conditions when evaluating the acoustic environment in the nursing homes. Moreover, the selected case should have a large sample size, which can improve the questionnaire distribution and increase the reliability of the data. The selected cases should be exposed to high noise in urban areas, so as to reflect real acoustic problems. Through visits, cases with a small number of people and cases with a better sound environment in the outer suburbs were excluded. Finally, the Aixin Nursing Home with a large number of people, large scale, and perfect facilities in the urban area was selected as the research site.

**Figure 1:**
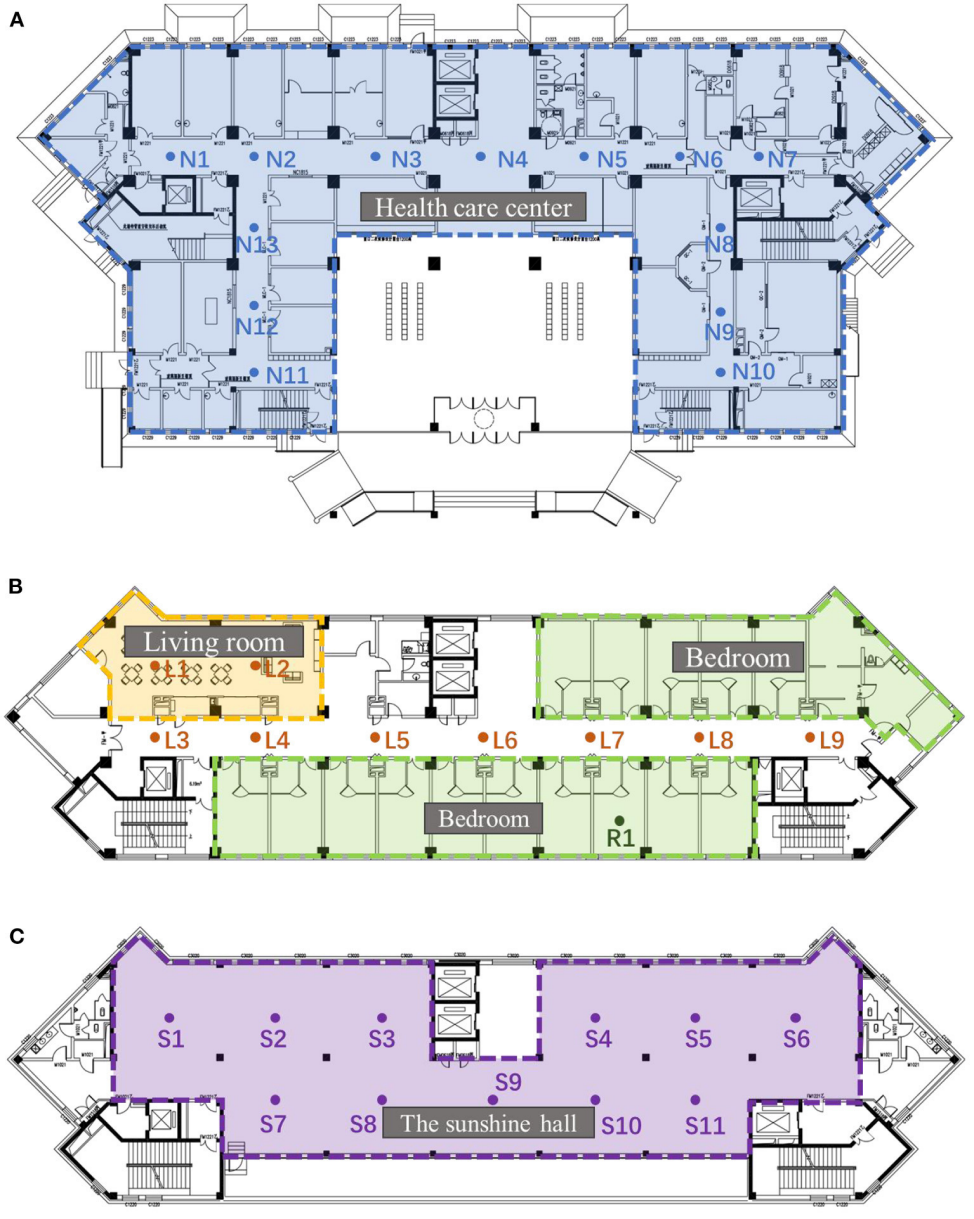
Layout of different space **(A)** Healthcare spaces layout **(B)** Living space **(C)** Sunshine Hall.

The layout and indoor public space of the selected research area were typical layout patterns of nursing homes in Harbin. Moreover, this research site is also the most well-known nursing home serving the most customers in Harbin. Its nursing building covers a total area of 25,200 square meters and accommodates 596 beds with a total of 19 floors. Floors 1–3 are for medical and health care functions, providing daily medical consultation, physical examination, physical therapy, rehabilitation, and other services for the elderly. Floors 4–18 are designated to be the elderly living quarters, mainly double-bedrooms. Each floor is equipped with unit living space. The 19th floor is the sunshine hall designed to provide a public indoor activity place for the elderly (Figure 1). According to the different acoustic environments and behavior patterns, the test site was divided into two types of spaces: public space, including sunshine hall, unit living space, and corridor of health care center; and private space, including the bedroom. The tests were conducted at fixed points, and the behavior patterns of the elderly were captured by surveillance cameras in public spaces.

### SPL and RT Measurement

According to previous studies, SPL and RT are the main factors affecting human sound perception (Tavossi, 2003). When the indoor environmental SPL is 65dB (A), it will affect human health and produce cognitive issues (Berglund et al., 1999; Stansfeld and Matheson, 2003). In this paper, the SPL and RT in different spaces of the conservation house were selected as the factors to evaluate the indoor sound environment. The test instrument was arranged in a network format. This study focuses on the influence of existing environmental sound sources on space. Therefore, no fixed sound source points were set. The distribution of measured points is illustrated in Figure 1.

The SPL was measured from 4 A.M. to 8 P.M. when the space was in use during winter in 2020. During each measurement, the window was closed without air-conditioning. The 801 sound level meters were set to slow mode and recorded A-Weighting Leq every 10 s. To avoid the variability of the sound sources, an average of 10 SPL measurements per hour at each measuring point on the same day was taken (Zannin and Marcon, 2007; Meissner, 2008). To study the impact of different types of activities of the residents on the evaluation of the acoustic environment, the sound lever meter took instant readings every 10 s after completion of each questionnaire. At each measurement point, measurements were made 10 times to obviate sound source variability. A total of 1 min of data was obtained from each survey position (Stansfeld and Matheson, 2003). The mean value was calculated to obtain the corresponding SPL (Zahorik, 2002; Meng et al., 2017).

The RT was tested at night with the door closed and devoid of occupants, and an OS002 omnidirectional instrument was used to playback white noise at the measuring point in Figure 1. After stabilization, the sound source was turned off and 30 dB (A) (T_30_) [after extrapolation to 60 dB (A)] was recorded (Bautmans et al., 2007). For the large space in Figure 1C, T_30_ is used instead of T_60_ for calculation. The test standard was ISO3382 (Barron, 2005).

### Acoustic Comfort Survey

According to the preliminary interview survey, it was found that the elderly in nursing homes may have a polarization trend in their irritability to sounds. Moreover, loneliness is common among the elderly, especially among those who are widowed. Literature analysis shows that there may be a certain relationship between the sound preferences of the elderly and their physical and mental conditions. In order to clarify the perception of the elderly in a nursing home on the acoustic environment, trap questions were set. The comfort of the acoustic environment in nursing homes might be related to the following factors:

1. Gender, age, and other social factors.
2. Loneliness and other psychological parameters.
3. Hearing, physical, and other physiological parameters.
4. Luminous environment, humidity, and other environmental factors.
5. Properties of sound field such as sound pressure level (SPL) and sound source in the building.

In the questionnaire, the attitudes of the elderly were measured using a five-point Likert-type scale (Table 1), which has been widely used in survey research on the environmental effects of subjective comfort (Sanchez et al., 2017; Liu and Kang, 2018). A total of 348 elderly people were surveyed from September 2020 to March 2021. The reliability coefficient of the questionnaire was estimated at 0.87 (Cronbach's alpha). The KMO coefficient was 0.705, and Bartlett spherical test results were significant (*P* < 0.001).

**Table 1:** The content of the questionnaire.

**Category**	**Questions**	**Scale**
Background	1. Gender, age 2. Education level 3. Marital status, offspring 4. Income (per month) 5. Hearing status 6. Sleeping status	1. very good 2. good 3. normal 4. bad 5. very bad 1. very good 2. good 3. normal 4. bad 5. very bad
ULS-8 Loneliness rating	1.Lack of company 2. There was no one to turn to for help 3. I am a person who likes to make friends 4. I feel left out 5. I felt alienated from other people 6. I feel sad because I have so little society 7. I can find someone to accompany me when I need them 8. I feel lonely	1. Never 2. seldom 3. Sometimes 4. all the time
PHQ-9 Depression	1. Work with little enthusiasm or interest 2. Feeling down, depressed, or hopeless 3. Difficulty falling asleep, restlessness, or excessive sleep 4. Feeling tired or without energy 5. Feeling you're a failure, or you've let yourself or family down 6. Have trouble focusing on things 7. Move or speak slowly enough for others to notice? Or just the opposite, fidgety or fidgeting and moving more than usual 8. Loss of appetite or eating too much 9. Suicide or want to harm yourself	0: never 1: several days 2: half 3: always
ADL	Use of public vehicles. Walk. Cooking. Do housework. Take medicine. Dining. Garb. Wash clothes. Bathing. Shopping. Ring up	0: yes 1: slightly difficult 2: need help 3: no
Satisfaction with the living environment	Sound environment Luminous environment Thermal environment Humid environment Smell	Very comfortable: 5 Comfortable: 4 Generally: 3 Uncomfortable: 2 Very uncomfortable: 1
Degree of sound preference	Voice Musical sound Sound of snoring Ring tones TV sound Washing machine sound Sound of rain and wind Chirp Traffic noise Construction noise Decoration noise	Enjoy: 5 Like: 4 Generally: 3 Dislike: 2 Hate: 1

To ensure that the participants had the appropriate physiological and psychological status to enroll in the study, the questionnaires were taken using a one-to-one method and completed within 5 min, and at least 10 interviews were conducted at each survey point. The residents who participated in the survey were considered qualified according to the frailty scales proposed by Rockwood et al. (2005), belonging to the scale range of 1–4. A total of 348 questionnaires were distributed, of which 329 were found to be valid.

### Statistics and Analysis

SPSS 20.0 was used to establish a database of the subjective and objective results (Zhang et al., 2018). One of the main research contents of the questionnaire was the influencing factors of the evaluation of the comfort level of the acoustic environment. Correlation between data was mainly calculated through correlation analysis. Table 2 lists correlation analysis methods and the choice behind different types of data.

**Table 2:** Correlation analysis and calculation methods of different independent variables and dependent variables.

**Independent variable**	**Dependent variable**	**Statistical mode**	**Index**	**Reasons for choosing mode and index**
Gend	Evaluation of acoustic environment comfort level	Independent-samples *t*-test	Mean difference	Dichotomous variables - Ordinal variables
Marital status				
Income level		Crosstabs	Crammer's V	Classified variables - Ordinal variables
Age		Bivariate correlation	Pearson correlation coefficient	Interval variables - Ordinalvariables
Length of stay				
The number of children				
The activity of daily living (ADL)				
Loneliness index				
Depression rating scale				
SPL				
Hearing status		Crosstabs	Camma correlation coefficient	Ordinal variables - ordinal variables
Sleep status				
Other				
SPL at different locations at the same time		Bivariate correlation	Pearson correlation coefficient	Interval variables-Interval variables

## Results

### Space-Time Distribution of Sound Source and Soundscape

#### Acoustics Characteristics of Different Functional Spaces

**Figure 2:**
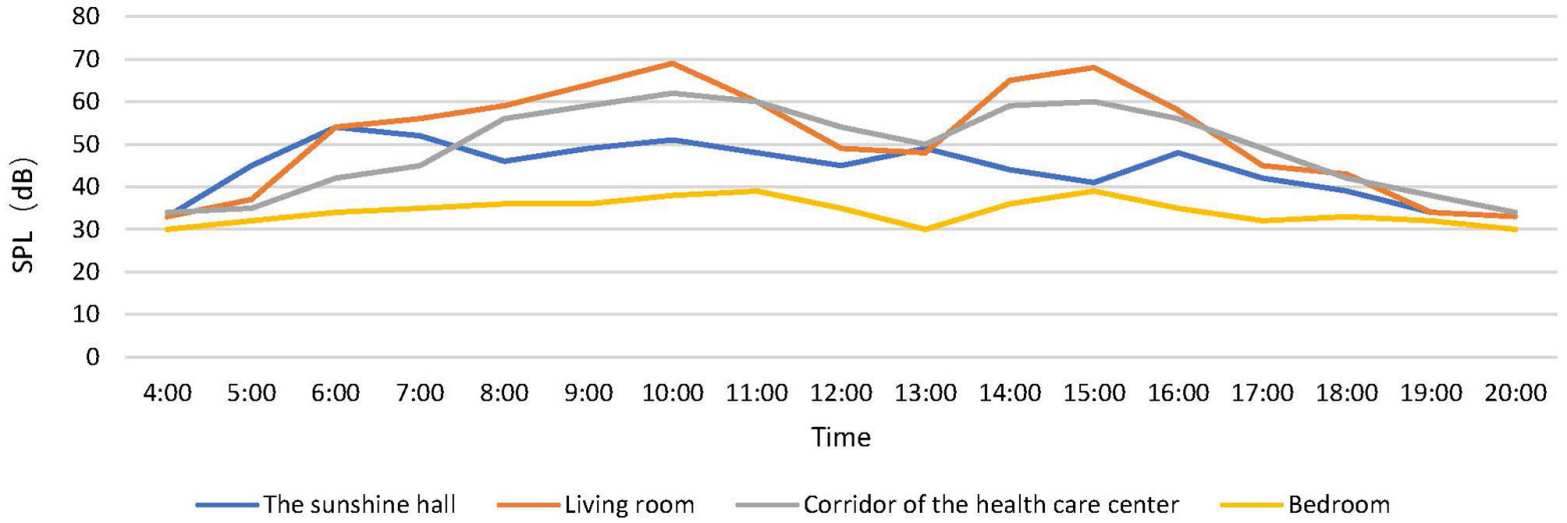
Daily SPL(A) distribution in four kinds of spaces.

According to the measured data, the variation of SPL in different spaces in the nursing building with time is depicted in Figure 2. The SPL in the living space of the unit was higher, and two peaks were noted. The highest one reached more than 60 dB(A). The average SPL in the Sunshine Hall was 45dB (A), and there was a peak between 5 A.M. and 7 A.M., followed by a slight increase at noon and at night. The SPL in the bedrooms of the elderly remained roughly between 30 and 40 dB (A) throughout the day and were slightly higher in the afternoon than in the morning and evening. The SPL of the corridor of the health care center was high during the working hours but did not exceed 60 dB, and it stayed at 32 dB in the morning and evening.

**Figure 3:**
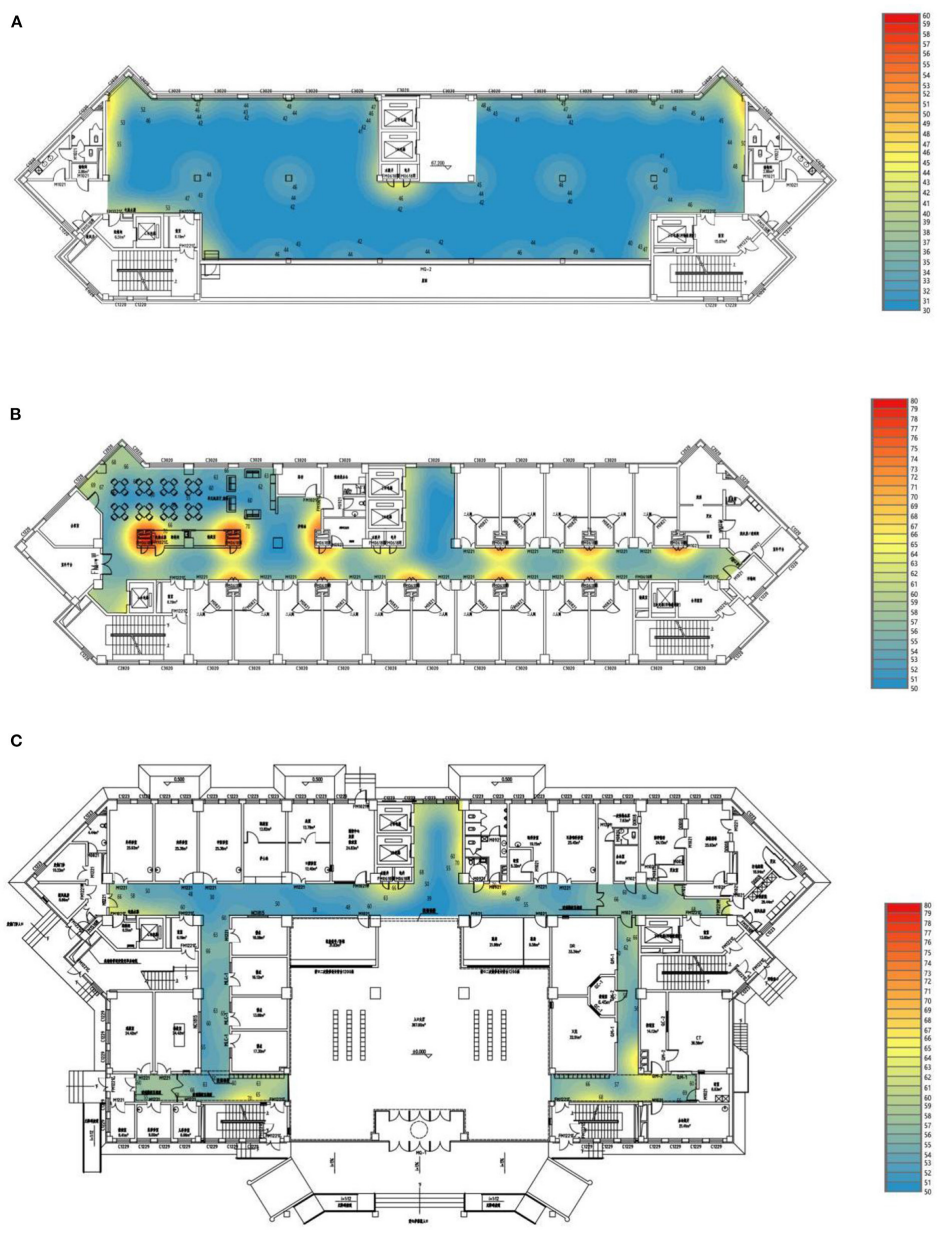
SPL distribution of different space **(A)** sunshine hall **(B)** living room **(C)** corridor of the health care center.

The distribution of SPL in specific places in several main spaces was analyzed at 3 P.M. when there were a lot of personnel activities, as shown in Figure 3. The SPL distribution of the sunshine hall was relatively uniform due to the large area of the hall, and the number of users was relatively scattered. The distribution of SPL in the living space of the units and corridors of the health care center was regionally enhanced. The areas with higher SPL were located near the main vertical traffic and near the entrances and exits of functional rooms.

**Figure 4:**
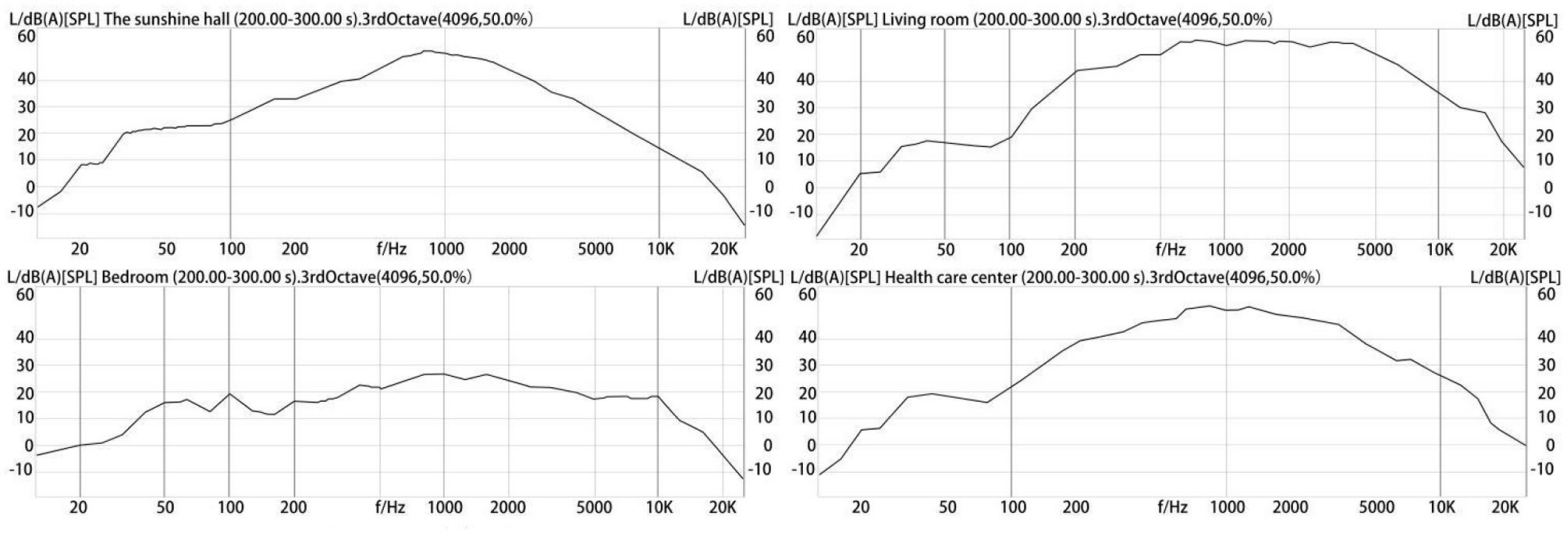
Sound spectrum analysis of the nursing home.

The Artemis S was used to carry out the spectrum analysis of sound samples. Figure 4 shows the sound spectrum analysis of the four kinds of public spaces selected. The spectrum measurement range in the figure was 20 Hz−20 kHz, and the image format was recorded with a continuous spectrum of 1/3 octaves.

It can be inferred from the figure that the frequency of noise in the sunshine hall was mainly concentrated between 250 Hz and 4 kHz, which was also the main frequency range of human language voice frequency distribution. This means the main component of the background noise in the sunshine hall was a human voice. The SPL in the bedrooms of the elderly was generally lower than 30dB (A), indicating that the indoor space of the elderly was a relatively quiet space which was due to the slow movement of the elderly. The unit living space was noisy, with a frequency ranging from 200 Hz to 10 kHz, indicating that the space was not only filled with human voice but also contained a certain high-frequency noise. The spectrum of the corridor of the health care center shows that in addition to human speech, there was a certain amount of high-frequency noise in this space, but it was slightly quieter than the unit living space.

#### RT of Different Functional Spaces

Reverberation time is one of the important indicators in indoor acoustic design. It is the time required from the moment when the sound source stops sounding to the attenuation of the sound energy density of 60dB (A) after the indoor sound field reached a stable state. Excessive RT in a room will cause the sound to lack clarity, whereas an excessively short RT will cause the sound to be dry and lack vitality. Table 3 shows a list of RTs at different frequencies for the sun hall, unit living spaces, bedrooms, and corridors of the health care center.

**Table 3:** RT at different frequencies in public space.

**Type**	**125 HZ(s)**	**250 HZ(s)**	**500 HZ(s)**	**1,000 HZ(s)**	**2,000 HZ(s)**	**4,000 HZ(s)**
The sunshine hall	1.42	1.87	2.32	2.35	2.55	2.16
Living room	2.03	2.35	2.25	2.15	2.13	1.95
Bedroom	1.33	1.25	1.22	1.13	1.02	0.98
Health care center (Corridor)	1.85	2.97	2.68	2.13	2.05	1.76

It can be inferred from the table that the RT in the sunshine hall was relatively long, and the average RT value at the frequency of 500–1,000 Hz was 2.33 s, which was well-beyond the recommended value of the reverberation time in the normal hall. The reason for this phenomenon is that the area of the sunshine hall is larger and the story height is higher, such a large volume and without any sound absorption device will seriously affect the sound transmission in the hall. In contrast, the RT in the bedrooms of the elderly is ideal. The average RT value of the unit living space and corridor of the health care center was 2.2 and 2.4 s, respectively, at the frequency of 500–1,000 Hz. Therefore, some measures should be taken to control the acoustic parameters such as the RT within the ideal range.

### Sound Preference and Sound Comfort of the Elderly

#### The Influence of the Background of the Respondents on the Comfort of the Acoustic Environment

In the questionnaire, the major factors that influence the evaluation of acoustic environment comfort include gender, marital status, income level, age, hearing condition, and sleep condition of the respondents. In the actual survey, there were only two types of the marital status of the elderly in nursing homes: married and widowed. Therefore, both marital status and gender belong to the two categorical variables. As illustrated in Table 4, an independent sample *t*-test was conducted for gender and marital status to assess any difference in the evaluation of the acoustic environment comfort level of the elderly under different classifications. It can be inferred from the table that gender factors had no significant influence on the evaluation of acoustic environment comfort of nursing homes. However, marital status had a significant influence on the evaluation of acoustic environment comfort, and the average value of the married elderly (3.44) exceeded that of the widowed elderly (3.29).

**Table 4:** Correlation analysis of evaluation of acoustic environment comfort level and social background.

**Dependent variable**	**Independent variable**	**Statistical model**	**Correlation analysis**							
				**F**	**Si g**.	**t**	**df**	**Sig**.	**Mean difference**	**Standard error**
Evaluation of acoustic environment comfort level	Gender	Independent-samples *T*-test	variance is equal	0.358	0.546	1.676	111	0.094	0.361	0.215
			variance is not equal	–	–	1.681	107.76	0.093	0.361	0.215
	Marital status		variance is equal	0.485	0.486	0.664	111	0.504	0.141	0.212
			variance is not equal	–	–	0.668	108.13	0.501	0.141	0.211
	Income level	Crosstabs		**Value**				**Approximation Sig**.		
			Scalar	ϕ	0.473^*^			0.000		
				Cramer V	0.336^**^			0.000		
			N in the valid case					113		
			**Pearson**				**Sig. (bilateral)**			
	Age	Bivariate correlation	0.356^**^				0.000			
	Number of children		0.164				0.075			
	Time of residence		−0.78				0.415			
		Crosstabs	**Value**	**Asymptotic standard error**			**Approximation T**		**Approximation Sig**.	
	Hearing status		−0.515	0.135			−3.315		0.001	
	Sleep status		0.273	0.116			2.226		0.028	

** *indicates that the two-tailed test is significant at the level of 0.01, and*

* *indicates that it is significant at the level of 0.05*.

In the actual survey, the actual income of the elderly was not clearly compiled, and the income of the elderly was related to the income of their children. Therefore, the income factor was set as a qualitative variable, which was divided into three grades: high, middle, and low. Hence, Crammer's V coefficient in the SPSS cross table was used to calculate the correlation coefficient between this variable and the evaluation of the comfort level of the acoustic environment. As inferred from Table 4, the correlation between the comfort level of the acoustic environment and income was 0.336^**^(*P* < 0.01). However, since Cramer's V coefficient is a symmetric measurement, the coefficient had no positive or negative points. Through the analysis of the mean value of the data, it was found that the mean value of the evaluation of the low-income elderly was 3.45^**^(*P* < 0.01), the middle income was 3.33^**^(*P* < 0.01), and the high income was 3.27^**^(*P* < 0.01), indicating that with the increase of income, the evaluation the elderly on the comfort of the acoustic environment showed a downward trend.

Time of residence, age, and a number of children are all continuous quantitative variables, and a Pearson correlation coefficient was adopted for calculation. As inferred from Table 4, there was a significant correlation between age and comfort evaluation of acoustic environment, with a correlation coefficient of 0.356^**^(*P* < 0.01), indicating that with the increase of age, the evaluation of the elderly on the comfort level of acoustic environment exhibited an increasing trend, and the elderly had a higher tolerance to the acoustic environment than the younger ones. Moreover, the *P*-values of the significance test of the number of children and the time of stay all exceeded 0.05, indicating an insignificant correlation between these two variables and the comfort level of the acoustic environment.

It can be inferred from Table 4 that the correlation between the acoustic environment comfort and the hearing condition was−0.515^**^(*P* < 0.01), and the correlation between the acoustic environment comfort and sleep quality was 0.273^*^(*P* < 0.05). The results showed that older people with better hearing conditions had a worse evaluation of the comfort level of the acoustic environment, whereas the older people with better sleep conditions had a higher evaluation of the comfort level of the acoustic environment.

#### Influence of Physical and Mental Health Indexes on Acoustic Environment Comfort

Physical and mental health indexes include loneliness index (LI), depression rating scale (DRS), activities of daily living (ADL). As these three variables are equidistant quantitative variables, a Pearson correlation coefficient was used to calculate correlation analysis. The specific calculation results are listed in Table 5.

**Table 5:** Correlation analysis of loneliness index, depression degree, and activity ability with acoustic environment comfort.

	**Correlation coefficient/Significance level**			
	**Acoustic environment evaluation**	**LI**	**DRS**	**ADL**
Acoustic environment evaluation	1	−0.627/0.000(^**^)	−0.532/0.000(^**^)	0.355/0.000(^**^)
LI	–	1	0.844/0.000(^**^)	−0.389/0.000(^**^)
DRS	–	–	1	−0.299/0.022(^*^)
ADL	–	–	–	1

** *Indicates that the two-tailed test is significant at the level of 0.01, and*

* *indicates that it is significant at the level of 0.05*.

As Table 5 shows, LI, DRS, ADL were all significantly correlated with the evaluation of comfort level of the acoustic environment, and the correlation coefficients were −0.627^**^(*P* < 0.01), −0.532^**^(*P* < 0.01), and 0.355^*^(*P* < 0.05), respectively. It can be concluded that the elderly with greater loneliness and depression had a worse evaluation of the acoustic environment, whereas the elderly with better physical ability have a higher evaluation of the acoustic environment. Notably, the correlation coefficient between LI and DRS was as high as 0.844^**^(*P* < 0.01). This showcases the strong correlation between the two variables. Modern psychology believes that loneliness and depression represent two different psychological states, but also stem from different reasons. Therefore, the two psychological states are discussed as independent factors, instead of being combined into one variable.

#### Correlation Analysis of Different Types of Environmental Comfort

Numerous pieces of literature show that there is a correlation between human perception of the acoustic environment and other environmental indicators (Yu and Kang, 2010; Meng and Kang, 2013; Wu et al., 2020b). This study lists illumination, temperature, RH, smell, and the overall evaluation of six main sensory comfort evaluation indices in the questionnaire. Since each variable is a 5-level scale, the gamma coefficient was used to calculate the correlation coefficient between other environmental comfort evaluations and acoustic environmental comfort evaluation. The specific parameters are listed in Table 6.

**Table 6:** Correlation analysis of different types of environmental comfort.

	**Parameter statistics of sensory comfort evaluation**					**Correlation analysis of other environmental factors and comfort of the acoustic environment**			
	**Mean value**	**Standard deviation**	**The standard error**	**Kurtosis**	**Skewness**	**Value**	**Asymptotic standard error**	**Approximation T**	**Approximation Sig**.
Acoustic environment	3.36	1.120	0.103	−0.875	−0.080	-	-	-	-
Luminous environment	3.43	1.042	0.098	−0.475	−0.230	0.419	0.146	2.591	0.010
Temperature	2.74	1.265	0.118	−0.946	0.204	0.232	0.117	2.226	0.025
RH	3.34	0.996	0.095	−0.242	−0.035	0.486	0.116	3.654	0.000
Smell	3.42	0.862	0.082	0.736	−0.559	0.303	0.153	1.863	0.062
Global assessment	3.26	0.882	0.085	0.694	0.049	0.515	0.143	3.285	0.001

As shown in Table 6, the evaluation of the elderly of the luminous environment was the highest at 3.43, followed by smell at 3.42, and acoustic environment comfort at 3.36. The average value of RH was 3.34, whereas temperature accounted for the lowest value at 2.74. The mean of the overall evaluation was 3.26, lower than the average except for temperature. The acoustic environment had the closest relationship with humidity, with the specific parameter value of 0.486^**^(*P* < 0.01), and the correlation coefficient between the acoustic environment and the luminous environment was 0.419^**^(*P* < 0.01). The correlation between acoustic environment and smell failed to pass the significance test. The correlation between the two could therefore not be proved. The correlation with temperature was 0.232^*^(*P* < 0.05). It can be inferred that the evaluation of the acoustic environment was correlated with all the environmental factors except smell. Moreover, the correlation coefficient between the evaluation of acoustic environment comfort and the overall environmental evaluation was very high, reaching 0.515^**^(*P* < 0.01), indicating that the evaluation of acoustic environment also affects the overall environmental evaluation of nursing homes to a large extent.

In this paper, 40 elderly people (10 people per function space) were randomly surveyed in various locations of the nursing home. They were surveyed regarding the sound comfort of the environment, and the sound in the acoustic environment was recorded at the same time. The SPL and Speech Transmission Index (STI) were calculated and compared with the subjective evaluation of sound comfort, and the relevant lists of sound environment comfort, SPL, and STI were obtained. There was a significant correlation between the sound environment comfort and the measured SPL. The higher the SPL was, the worse the comfort evaluation, and the correlation coefficient was −0.711^**^(*P* < 0.001). There was also a significant correlation between STI and comfort level, with a correlation coefficient of 0.755^**^(*P* < 0.001).

**Figure 5:**
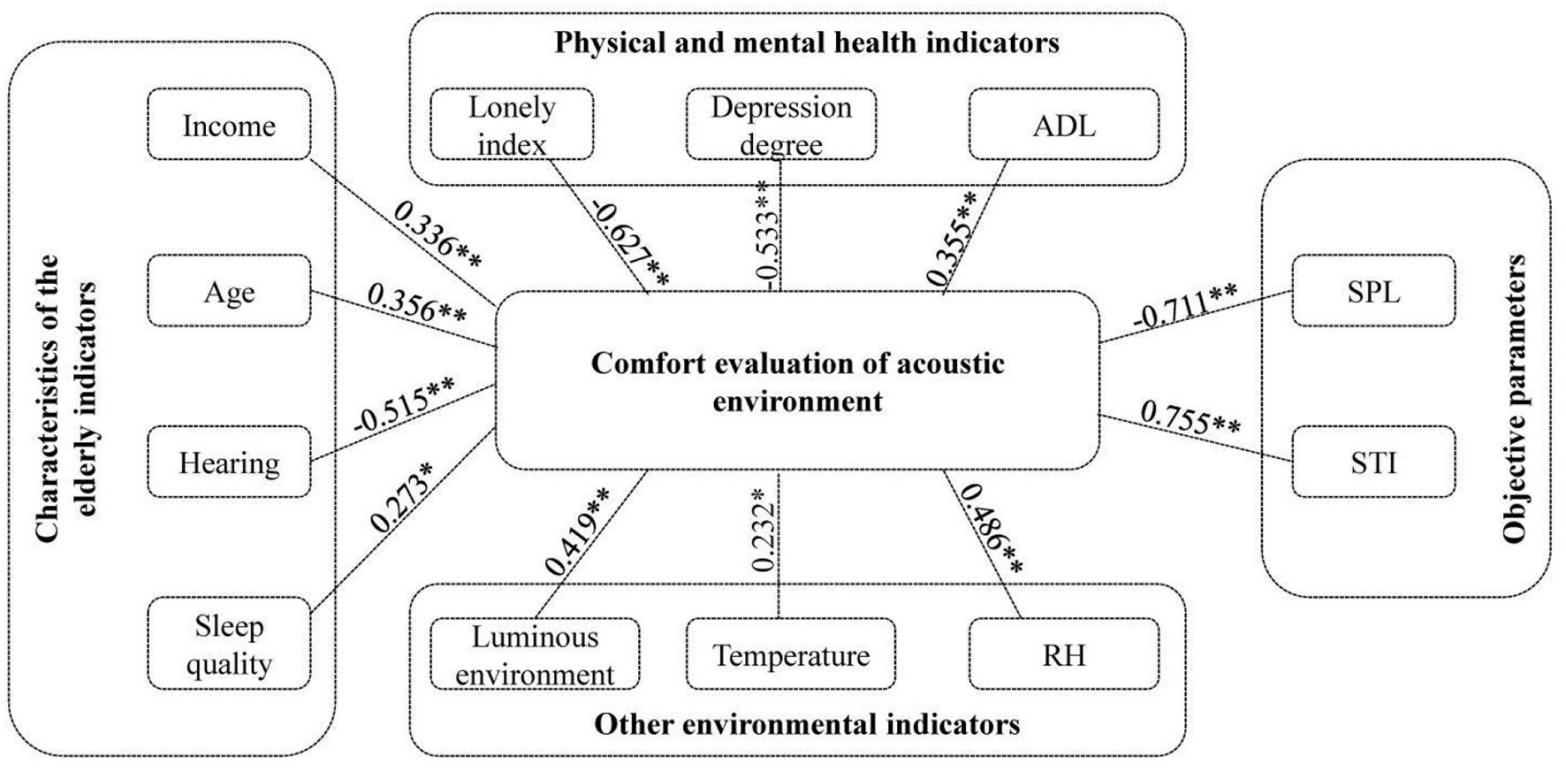
The relationship between different variables and comfort evaluation of acoustic environment. **indicates that the two-tailed test is significant at the level of 0.01, and *indicates that it is significant at the level of 0.05.

The results of the subjective and objective correlation analysis can be combined with the schematic diagram of the evaluation of acoustic environment comfort, as depicted in Figure 5, to yield a complete schematic diagram of the subjective and objective influence of acoustic environment comfort. The correlation coefficients in the table were decimals between 0 and 1. Although the magnitude of the correlation coefficient can indicate the strength of the correlation between data, it is the only representative of the correlation trend.

### Evaluation of the Sound Environment Based on the Sound Types and Sources

#### Correlation Between Acoustic Evaluation and Sound Source in Different Areas

The elderly people were asked about their subjective feelings when hearing different sound sources in different areas to assess the presence of a correlation between sound environment evaluations and sound types in different areas (Figure 1).

Column A in Table 7 lists the mean and standard deviation of the acoustic comfort evaluations of the elderly residents. Among them, the Sunshine hall was rated higher than all the other areas for all sound types, with an average close to 4. The elderly frequenting the area mainly enjoy the sunshine or practice sports. Moreover, vegetation is generally placed in the sunshine hall, and the pleasant environment improves the evaluation of the acoustic environment by the elderly, confirming Mu et al.'s study that a pleasant landscape or appealing visual scene can boost hearing comfort (Yu and Kang, 2010). Mechanical sounds were most annoying to the elderly, with a minimum rating of 1.73. The reason is that the sound of conversation and activities of the elderly has little influence on the surrounding area of the large space of the sunshine hall. However, the equipment room located on the roof had a greater impact. It can be inferred that the design of nursing homes should fully consider the behavioral pattern of the elderly and the influence of the acoustic environment, to arrange different functional areas.

**Table 7:** Correlation between sound sources and acoustic comfort.

**Name of area**	**Sound types**	**Sound sources**	**A: Average value/variance**	**B: Correlation**
				**Coefficient/*p*-value**
Sunshine hall	Activity sound		3.556/1.031	0.186/000(^***^)
		Walking sounds	2.925/1.884	0.16/0.064
		Sport sounds	2.842/1.636	0.425/000(^***^)
	Background music	Sound of rain and wind	3.63/0.925	0.222/0.105
	Mechanical sound	Device running sound	1.733/1.277	0.343/0.09(^*^)
	Speech sound	Talking sounds	2.761/1.414	0.577/0.00(^***^)
Corridor	Activity sound		2.764/1.244	0.325/0.022(^*^)
		Walking sounds	3.196/1.032	0.314/0.015(^*^)
		Seat and table moving sounds	2.296/1.434	0.222/0.087
		Playing card sounds	2.048/1.785	0.151/0.00(^**^)
	Background music	Background music	3.42/1.344	0.425/0.02(^**^)
	Foreground music		2.297/0.999	0.408/0.00(^**^)
		Music from electronic devices	3.024/1.597	0.259/0.001(^**^)
		Singing sounds	1.728/0.556	0.223/0.086
	Mechanical sound		2.452/1.245	0.420/0.02(^**^)
		Air conditioning sounds	2.471/1.286	0.057/0.655
		Trolley sounds	2.546/1.241	0.220/0.004(^**^)
	Speech sound		2.385/1.076	0.188/0.187
		Talking sounds of staff	1.965/0.636	0.225/0.08
		Talking sounds of onlookers	2.732/1.835	0.153/0.009(^*^)
Living room	Activity sound		3.061/1.345	0.595/0.000(^***^)
		Playing card sounds	3.261/1.702	0.341/0.005(^**^)
		Seat and table moving sounds	1.963/1.745	−0.024/0.816
		Dancing sounds	3.02/1.941	−0.035/0.719
		Chess sounds	2.944/1.223	0.334/0.001(^**^)
	Background music		2.78/1.638	0.495/0.001(^**^)
		Background music	3.171/1.445	0.451/0.000(^***^)
		TV sounds	2.261/1.778	−0.094/0.340
	Foreground music		1.542/1.402	0.539/0.000(^***^)
		Ring tones	1.747/1.585	0.268/0.002(^**^)
		Singing sounds	1.463/1.687	0.349/0.000(^***^)
	Mechanical sound		1.604/1.327	0.244/0.112
		Decoration noise	1.511/1.402	0.319/0.000(^***^)
		Construction noise	1.721/1.585	0.148/0.002(^**^)
	Speech sound		3.165/1.512	0.538/0.000(^***^)
		Talking sounds	3.645/1.644	0.215/0.000(^***^)
		Explanation sounds	2.868/1.675	−0.211/0.033(^*^)
		Talking sounds of staff	2.924/1.863	0.035/0.756
		Talking sounds of onlookers	2.932/1.828	0.097/0.324
Bedroom	Activity sound	Walking sounds	2.331/0.838	−0.038/0.713
	Background music	TV sounds	2.47/1.401	0.691/0.000(^***^)
	Foreground music	Chirp	2.14/1.043	0.358/0.061
	Mechanical sound	Washing machine sound	2.50/1.037	0.662/0.000(^***^)
	Speech sound		1.772/0.915	0.414/0.030(^*^)
		Talking on the phone sounds	1.442/1.402	0.539/0.000(^***^)
		Sound of snoring	1.947/1.585	0.268/0.002(^**^)

Due to the short time people spend in the corridor, the sound sources might be multidirectional, and the elderly have a reduced ability to capture sound. In this area, the respondents least liked the sound of talking and singing. Nevertheless, it had high voting regarding background music (3.42), and when voices were combined with background music, the ability of elderly residents to perceive and differentiate the sounds was affected. Therefore, the acoustic comfort of the elderly could be significantly improved by adding background music to the corridor.

The ratings for the living room's acoustic environment were low due to the poor overall sound environment. In this area, the most insufferable sound was renovation noise and construction noise garnering votes of 1.51, 1.72, respectively. Seat and table moving sounds, ring tones, and singing sounds were also shown to be insupportable. The most popular sound residents preferred was the background music. The reason for this result is that the elderly were engrossed in their activities, ignoring external activity and background sounds in the living room; high-frequency sounds could distract their attention and create discomfort.

The bedroom received the lowest overall evaluation of the acoustic environment. Talking on the phone sounds was the most unpopular sound source with the lowest voting of 1.44. Snoring sounds also disturbed the elderly. It's worth noting that in private spaces, other people's voices were the most unbearable sound source rather than activity sounds and mechanical sounds.

From Table 7, it can be concluded that the function of the building determines the sound source. For nursing homes, old people staying in a relatively narrow space (including the corridor and bedroom) require some background ambient sound and reduce the impact of human sounds. For large multifunctional spaces (including the hall and the living room), mechanical sounds have become the most intolerable sound source to the elderly. Besides, different sounds are mixed together, and when elderly people are unable to recognize and judge the content of sounds, they may become anxious, thereby impacting comfort levels.

#### Analysis on the Influencing Factors of Sound Preference of the Elderly

In this study, the 11 kinds of common sound sources that are summarized above were listed in the questionnaire scale. As there are numerous sound sources, a single analysis is of little significance. Therefore, this study conducted a factor analysis on 11 kinds of sound sources in nursing homes, seeking common characteristics and classification of different sound sources.

The KMO coefficient of the sound preference scale was 0.705, indicating the suitability of the data for factor analysis. The factor extraction method in this study was the iterative method based on principal component analysis, and the default factor extraction quantity in the calculation was based on the number of components extracted when the characteristic root exceeded 1. It can be inferred from Table 8 that a total of four factors were extracted according to the principle that the extraction feature root exceeded 1, and the cumulative contribution of the four factors to all 11 variables was 77.368%, indicating that the interpretation degree of the model was good.

**Table 8:** Factor analysis explained the total variance.

**Factor**	**Initial eigenvalue**			**Extract sum of squares load**			**Rotation sums of squared load**		
	**Sum**	**Variance %**	**Accumulation %**	**Sum**	**Variance %**	**Accumulation %**	**Sum**	**Variance %**	**Accumulation %**
1	4.883	40.712	40.712	4.876	40.698	40.698	3.485	29.082	29.086
2	1.742	14.523	55.234	1.739	14.521	55.232	2.586	21.608	50.692
3	1.619	13.514	68.745	1.612	13.515	68.752	2.010	2.019	67.459
4	1.038	8.612	77.371	1.036	8.613	77.371	1.179	1.192	77.368
5	0.858	7.168	84.542	–	–	–	–	–	–
6	0.556	4.645	89.213	–	–	–	–	–	–
7	0.402	3.341	92.545	–	–	–	–	–	–
8	0.283	2.335	94.886	–	–	–	–	–	–
9	0.245	2.012	96.875	–	–	–	–	–	–
10	0.164	1.335	98.223	–	–	–	–	–	–
11	0.152	1.278	99.512	–	–	–	–	–	–

The model of the factor load matrix was rotated by the orthogonal rotation method with Kaiser standardization. The rotated factor load matrix is illustrated in Table 9. To facilitate reading, the analysis software reordered and simplified the table, and coefficients <0.1 were suppressed and output in the table. The common factor 1 is mainly related to the sounds of people talking, music, snoring, ringtone, TV, and washing machine. These factors are closely related to the daily behaviors of people and are common sounds in the nursing home. Common factor 2 is mainly related to wind and rain sounds, insect and bird sounds. This common factor is the common natural sound outside the conservation house. Common factor 3 mainly includes traffic noise, construction noise, and indoor maintenance noise, belonging to the common public noise in the maintenance yard.

**Table 9:** Total variance explained by factor analysis.

	**Factor**				**Mean value and standard deviation of different sound sources**	
	**1**	**2**	**3**	**4**	**Mean value**	**Standard deviation**
Voice	0.826	−0.396	−0.133	−0.152	2.65	0.473
Musical sound	0.721	0.485	—	—	3.03	0.841
Sound of snoring	0.765	0.182	0.341	—	2.32	0.647
Ring tones	0.792	0.290	—	0.242	3.13	0.662
TV sound	0.739	0.352	0.196	0.206	3.05	1.012
Washing machine sound	0.425	0.296	0.193	—	2.38	0.572
Sound of rain and wind	0.186	0.935	—	—	3.42	0.691
Chirp	0.260	0.890	0.183	—	3.31	0.872
Traffic noise	0.469	0.335	0.658	—	2.16	0.776
Construction noise	—	0.241	0.795	0.335	1.73	0.706
Decoration noise	—	−0.203	0.829	—	1.48	0.643

As inferred from Table 9, the scores of natural sound sources such as wind and rain and insects and birds sound are relatively high, reaching 3.42 and 3.31. It shows that elderly people are generally willing to accept such voices. However, the score of outdoor noise such as vehicle driving is very low, especially the score of indoor maintenance sound is only 1.48, indicating that such noise must be avoided in the acoustic environment. In addition, older people also rated mobile phone ringtones, music and TV sounds more highly, indicating that these sounds can meet their needs.

## Discussion

Through a literature review, Mu et al. (2021) carried a field measurement and questionnaire in another nursing home in Harbin in 2021. Despite varying research directions, old people in the same function but different building types have different sound perceptions. In their research, the acoustic environment of the activity space is the most unbearable which clashes with this study. The perception of the same sound sources also differs in the elderly. By comparison, the layout of space and management mode was found to be the cause of the difference. In their case, the living room, activity space, and bedroom are mixed together on 1–3 floors, but separately located on 4–19 floors in our case. The spatial function determines the acoustic environment. Therefore, it should not only consider the sample size of the survey population but also consider the different layout cases in acoustic questionnaires.

There are significant differences in sound perception among the elderly from different social and life backgrounds. Gender factor had no significant influence on the acoustic environment comfort evaluation of nursing homes, whereby different case studies yielded varying outcomes. However, marital status had a significant effect on the comfort level of an acoustic environment which was similar to other studies. With the increase of income, the elderly people evaluation of the comfort of acoustic environment exhibited a downward trend and with the increase of age, the elderly people evaluation of the comfort of acoustic environment showcased an increasing trend, and the elderly have a higher tolerance to the acoustic environment than the younger ones. Moreover, the correlation between the number of children and the comfort level of the acoustic environment was negligible. The older people with better hearing conditions had worse evaluation on the comfort of the acoustic environment, whereas the older people with better sleep conditions had higher evaluation on the comfort of the acoustic environment.

This study presents some limitations. The main point is related to the generalizability of the results of the current study to other test sites and contexts. In particular, the perceived effectiveness of the acoustic correction interventions might be specific to the analyzed case study. However, the contribution of this work should be considered methodological and aimed at proposing a combined quantitative and qualitative approach in applications where only one is generally adopted.

## Conclusions

This research focuses on the evaluation of elderly residents of their acoustic comfort according to an on-site observation, sound measurements, and a questionnaire conducted in a public space in a nursing home in Harbin, China. On the basis of previous research, this study has been expanded to compare the acoustic perception of the elderly in public spaces and private rooms.

In general, the participants evaluated the acoustic environment in the case with a low rating. The SPL measurement found that the SPL in the unit living space in the nursing home was the highest and can exceed 65 dB (A) when using high frequency. The average SPL in the sunshine hall was 45 dB (A). The SPL in the bedrooms of the elderly remained roughly between 30 and 40dB (A) throughout the day and were slightly higher in the afternoon than in the morning and evening. The RT of the unit living space was 2.15 s at a frequency of 1,000 Hz, and the corridor of the health care center was 2.13 s, which exceeded the acceptable range. The evaluation of the acoustic environment also affected the overall environmental evaluation of the nursing home to a large extent. In terms of sound preferences, elderly people were generally willing to accept the sound of natural sound sources.

Acoustic comfort, preference, and noise levels are all affected by subjective perceptions, and loudness and clarity are affected by physical conditions. The evaluation of an acoustic environment is related to the social background and living background of the elderly. Marital status and income level are the main impact factor on the evaluation of the acoustic environment. Moreover, older people have a higher tolerance to the acoustic environment than younger ones. The layout of space and management mode of the nursing homes leads to different sound sources, altering the correlation with acoustic evaluation results in other cases. Future studies could improve the conciseness of the questionnaire more concisely and consider the visual factors and spatial function factors in an acoustic environment survey.

Regarding the acoustic design of nursing homes, the following strategies should be considered. In the cases investigated, the reverberation time of public space is too long, thereby affecting the semantic intelligibility of the elderly. The reverberation time should be controlled in public spaces and sound-absorbing materials should be in place. The addition of natural music to the public space can also make the residents more delighted. Moreover, humidifiers could be placed in public spaces to improve the acoustic comfort of the elderly. High-frequency noise should be controlled through the addition of perforated sound-absorbing panels in the bedroom. The loneliness index and ADL of the elderly markedly impact their acoustic perception. It is therefore recommended for nursing homes to organize more group activities in public spaces and enhance geriatric care toward the elderly suffering from behavioral difficulties.

## Data Availability Statement

The original contributions presented in the study are included in the article/supplementary material, further inquiries can be directed to the corresponding author/s.

## Ethics Statement

Ethical review and approval was not required for this study on human participants in accordance with local legislation and institutional requirements. The participants provided their written informed consent to participate in this study.

## Author Contributions

JZ: investigation, data collection, methodology, formal analysis, visualization, and writing–original draft. PC: supervision, investigation, and writing–review and editing. TL: resource, investigation, and software. All authors contributed to the article and approved the submitted version.

## Funding

This work is supported by Fundamental Research Funds for the Central Universities (2572021BK02) and Heilongjiang Province Philosophy and Social Science Research Planning Project (18SHB070).

## Conflict of Interest

The authors declare that the research was conducted in the absence of any commercial or financial relationships that could be construed as a potential conflict of interest.

## Publisher's Note

All claims expressed in this article are solely those of the authors and do not necessarily represent those of their affiliated organizations, or those of the publisher, the editors and the reviewers. Any product that may be evaluated in this article, or claim that may be made by its manufacturer, is not guaranteed or endorsed by the publisher.
